# Engineering Cup-Shaped Nanomotors for Promoting Cell Internalization and Synergistic Tumor Therapy

**DOI:** 10.34133/research.0623

**Published:** 2025-04-22

**Authors:** Ning Feng, Longteng Liang, Yufang Liu

**Affiliations:** ^1^ Henan Key Laboratory of Infrared Materials & Spectrum Measures and Applications, School of Physics, Henan Normal University, Xinxiang 453007, China.; ^2^ Institute of Physics, Henan Academy of Sciences, Zhengzhou 450046, China.

## Abstract

Self-propulsion enzymatic nanomotors have shown tremendous potential in the field of diagnostics. In a study led by Wang and coworkers, nanoenzyme-driven cup-shaped nanomotors were designed for enhanced cell penetration and synergistic photodynamic/thermal treatments under single near-infrared laser irradiation. By combining the concepts of self-propulsion enzymatic nanomotors and synergistic dual-modal therapy, this work provides a new idea and tool for the application of nanomotors in the biomedical field.

Nanocarriers have shown tremendous potential in the fields of drug delivery and diagnostics due to their unique advantages, such as high load capacity, extended system cycle time, and enhanced penetration and retention effects [1]. Nanocarrier-based light therapy highlights robust therapeutic capabilities because of its operational flexibility, noninvasive characteristics, low toxicity, and high spatial and temporal resolution [2]. Previous studies have shown that passive diffusion impairs the delivery efficiency of nanocarriers, resulting in poor biofilm penetration and nonspecific accumulation in the biological environment [3–5]. Therefore, it poses a great challenge to proactive searching and effective targeting of lesion location in the diagnosis process.

Recently, nanomotors have demonstrated the ability to convert external energy into mechanical motion [6]. By harnessing this property, nanomotors can transport nanocarriers to target cells, thereby enhancing cell uptake efficiency. Furthermore, the combination of nanomotors with therapeutic approaches represents an effective strategy for constructing active transport nanoplatforms that enable multimodal synergistic therapy [7]. This multifunctional approach can enhance both diagnostic accuracy and therapeutic efficacy.

Among various nanomotors, Janus nanomotors possess 2 distinct functional materials within a single particle, leading to asymmetric physical and chemical properties [8]. In biomedical applications, Janus nanomotors take advantage of an asymmetric structure for targeted delivery to specific cells. However, despite their substantial potential in cellular transport, there are still some problems to be solved, such as complex driving systems, inaccessible exogenous fuels, and the generation of cytotoxic by-products in biological environments. In June 2022, Wang’s group [9] published a fascinating work in *Research*, in which they designed a nanoenzyme-driven cup-shaped nanomotor that enables active cell targeting and collaborative photodynamic/thermal therapy through single near-infrared laser irradiation (Figure). This work sets up a model example for dual-modal cancer therapy, combining active cell targeting with collaborative photodynamic/thermal therapy.

**Figure.**
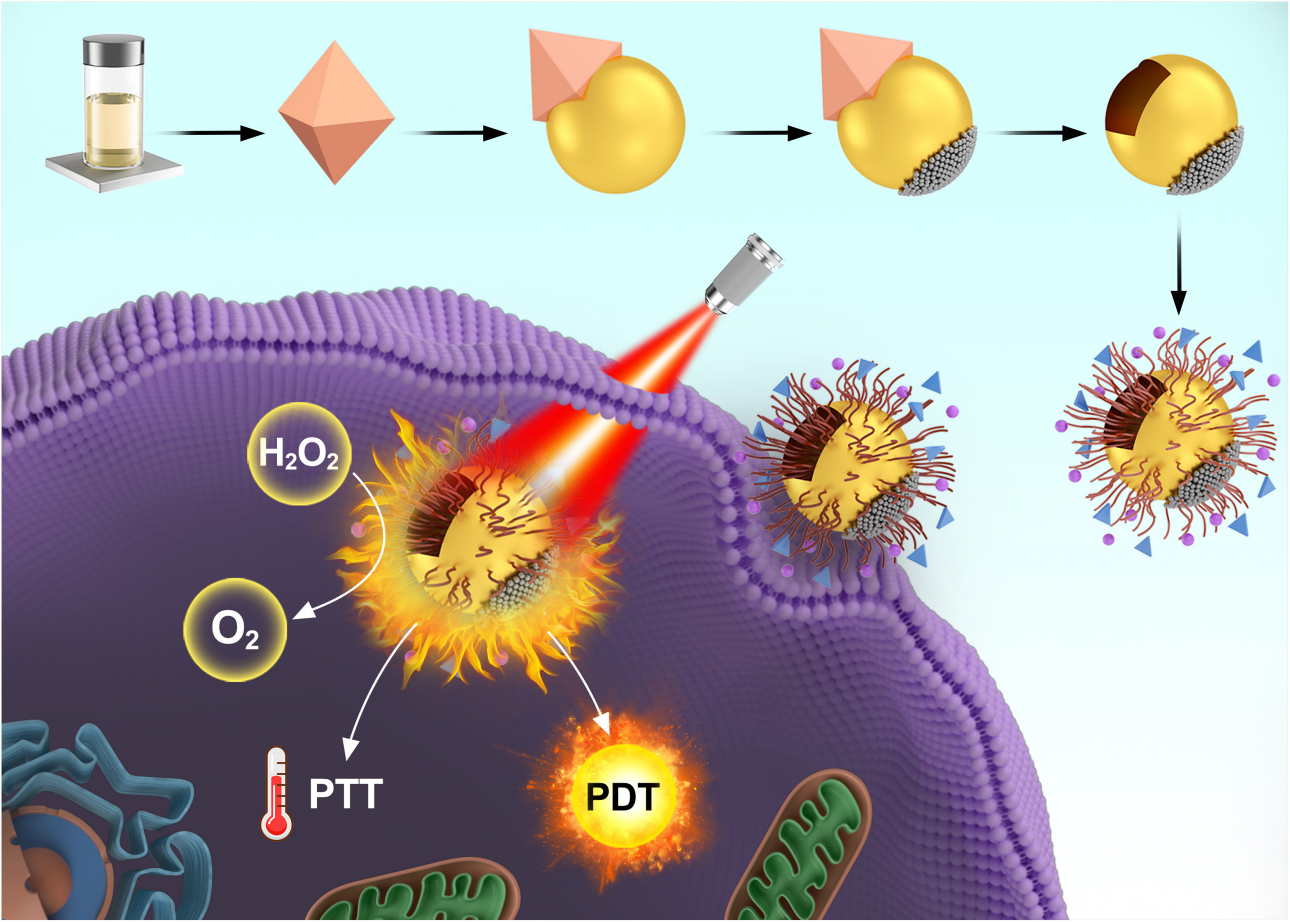
The nanoenzyme-driven cup-shaped nanomotor for collaborative photodynamic/thermal therapy. PTT, photothermal therapy; PDT; photodynamic therapy.

The cup-shaped nanomotors are composed of gold nanocups (GNCs) and platinum nanoparticles (PtNPs) asymmetrically decorated at the bottom. Platinum nanoparticles, possessing strong peroxidase-like activity, act as a continuous O_2_ generator. These nanomotors can drive the directed movement of nanoparticles by catalyzing the decomposition of endogenous H_2_O_2_. This enhanced directional movement leads to a broader diffusion area, which improves their search efficiency and drives them toward the target cell. This improved search efficiency is a substantial advantage over other types of nanomotors, as it enhances the targeting and delivery capabilities of the nanomotors. In contrast, many existing nanocarriers rely on external energy sources or passive diffusion, limiting their effectiveness in vivo. Furthermore, the cup-shaped structure provides a larger surface area for the modification of functional molecules, which is particularly important for loading therapeutic agents or targeting ligands. In this study, the decoration of indocyanine green (ICG) on gold nanocups–platinum (GNCs-Pt) enables efficient photodynamic reactions by fully utilizing the produced O_2_. Moreover, when decorated with ICG and transferrin, GNCs-Pt-ICG/Tf exhibits a wider diffusion area and faster speed on the HepG2 cell membrane, improving the cell uptake efficiency. This finding highlights the importance of surface modification in optimizing the delivery and efficacy of therapeutic agents.

Inspired by its remarkable ability to generate O_2_, this cup-shaped nanomotor can be utilized as an active nanocarrier for synergistic photodynamic/thermal treatments under single near-infrared laser irradiation. The generated O_2_ accelerates the photodynamic reaction, effectively overcoming the limitation of the tumor hypoxic environment [10]. Moreover, the photothermal conversion capability of nanomotors enables photothermal therapy (PTT). As a result of the dual-modal therapy, the cell mortality rate is substantially higher than that of the single-treatment mode group, suggesting the effectiveness of the synergistic therapy. The combination of photodynamic therapy (PDT) and PTT offers a powerful strategy to enhance the efficacy of cancer treatment.

Since the publication of the original study by Wang’s group, several marked advancements have been made in the field of cup-shaped nanomotors. The cup-shaped nanomotors, characterized by their unique structure, have demonstrated robust self-propulsion capabilities. This inherent property allows them to effectively penetrate cells, which is a key factor for biomedical applications [11]. Even more exciting, the integration of nanomotors with emerging technologies, such as gene therapy, drug delivery, and immunotherapy, offers novel applications in the therapeutic field [12,13]. For instance, a cup-shaped nanomotor-driven targeting chimera has been ingeniously designed to achieve effective internalization and degradation of extracellular proteins [14].

Compared to other types of nanomotors, cup-shaped nanomotors offer several advantages: (a) The cup-shaped structure generates an asymmetric propulsive force, which is beneficial for expanding the diffusion area and effectively recognizing biological targets. (b) The cup-shaped structure provides a larger surface area for functional molecules, allowing for the attachment of more targeting ligands and therapeutic agents, thereby enhancing both targeting and therapeutic capabilities. (c) The cup-shaped nanomotor locally produces O_2_, breaking the limitation of the hypoxic tumor microenvironment in PDT therapy. Additionally, the active diffusion behavior also brings nanomotors closer to O_2_, expanding the effective diffusion distance of O_2_, thereby promoting PDT.

Although cup-shaped nanomotors show various advantages in biomedical applications, there are still several issues in practical application. First, while cup-shaped nanomotors are prepared by a facile bottom-up approach, the preparation process requires stringent condition control, and reproducibility is difficult to guarantee, thereby limiting its widespread application in actual production. Second, H_2_O_2_-driven nanomotors rely on endogenous H_2_O_2_ for driving. However, the concentration of H_2_O_2_ in tumor cells is different, potentially leading to unstable motion performance. Third, although the designed nanomotors exhibit lower cell toxicity in short-term in vitro cell experiments, the potential long-term toxicity in vivo remains unknown. Furthermore, nanomaterials may accumulate in the body and cause long-term damage to normal tissues and organs, necessitating further in vivo studies for evaluation.
